# Choroidal Metastasis from Follicular Cell Thyroid Carcinoma Masquerading as Circumscribed Choroidal Haemangioma

**DOI:** 10.1155/2014/251817

**Published:** 2014-03-11

**Authors:** V. P. Papastefanou, A. K. Arora, J. L. Hungerford, V. M. L. Cohen

**Affiliations:** Ocular Oncology Service, St Bartholomew's and Moorfields Eye Hospital, London EC1A 7BE, UK

## Abstract

Choroidal metastases from follicular thyroid carcinoma are uncommon and usually present as an amelanotic lesion against a background of known systemic disease. We present the case of a 56-year-old woman with a thyroid metastatic focus with unusual clinical presentation, systemic involvement, and early response to systemic treatment. A review of the literature accompanies this case presentation.

## 1. Case Presentation

**Figure 1:**
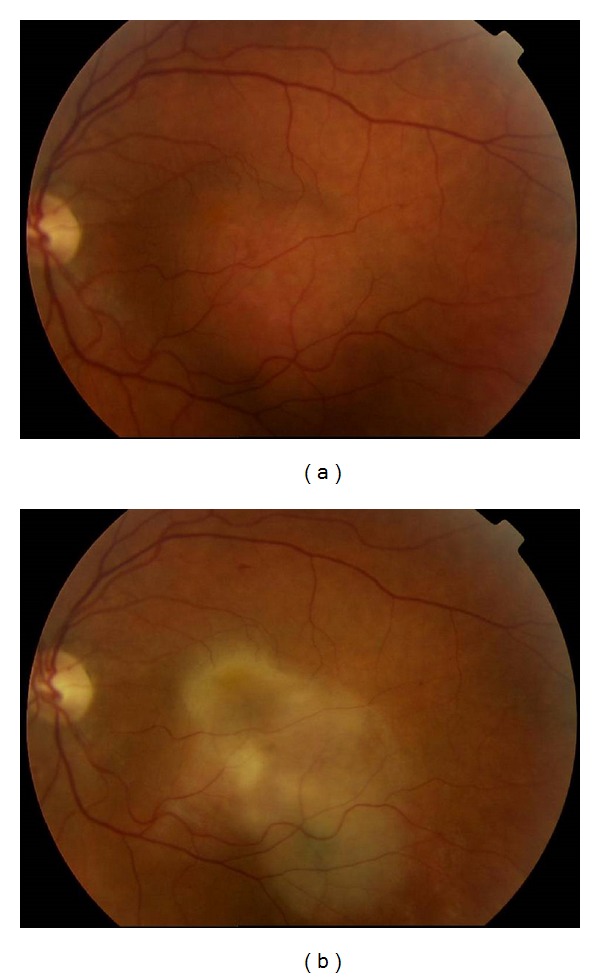
(a) Pale orange lesion involving the macula and extending inferotemporally before treatment. Clinical appearance is consistent with a circumscribed choroidal haemangioma. (b) After four months a pale, fibrotic scar has developed in the site of the original lesion following treatment.

A 56-year-old lady was referred to the Ocular Oncology Service with a seven-year history of deteriorating visual acuity in the left eye. Patient had a 15-year history of sarcoidosis associated with recurrent uveitis. Best-corrected visual acuity was at 6/36 OS 6/6 OD. At presentation there was no active uveitis. Posterior segment examination revealed an elevated lesion inferotemporally to the macula of the left eye measuring 6.5 × 6.8 mm that was pale orange in colour and difficult to discern clinically from the surrounding retina (Figure 1(a)). A fundus fluorescein angiogram indicated early hyperfluorescence. B-scan ultrasound demonstrated a dome-shaped lesion with high internal reflectivity measuring 2.7 mm in elevation. A Doppler B-scan ultrasound indicated the presence of internal blood flow. Clinical appearance at presentation was typical of a circumscribed choroidal haemangioma although a metastatic deposit remained within the differential diagnosis. No ocular treatment was performed but review was arranged in 3 months pending ongoing investigations for a thyroid gland mass discovered on a routine CT scan of the neck and chest.

Thyroid gland biopsy proved the mass was a follicular cell carcinoma of the thyroid and systemic staging revealed stage 4 disease due to the presence of bone metastases in the left iliac crest and in the right femur, the latter causing a pathologic fracture. Patient underwent total thyroidectomy. Excision was incomplete and patient received 2 initial sessions of adjuvant treatment of radioactive iodine (^131^I) therapy (4.9 and 5.8 GBq, resp.). The pathologic fracture was managed with internal fixation and adjuvant external beam radiotherapy (EBRT) with 35 Gy at 15 sessions in the iliac crest and 20 Gy in the femur.

Following radioactive treatment of the thyroid gland the visual acuity reduced to counting fingers. Fundus examination indicated a dramatic change in the appearance of the lesion from an orange, dome-shaped elevated mass to a flat fibrosed scar of pale-greenish colour (Figure 1(b)). This change was attributed to radioactive iodine uptake and prompted the diagnosis of a secondary metastatic lesion masquerading as a choroidal haemangioma.

Over the next five years the choroidal scar remained stable; no other ocular metastases were diagnosed, but the patient developed pulmonary metastases and a subcutaneous sternal nodule. She received 9 additional sessions of ^131^I (2.9–9.5 GBq) and two more sessions of EBRT in the nodule. She also followed monthly pamidronate infusions.

## 2. Discussion

Choroidal metastases are the most common intraocular tumour, with an estimated incidence of 20,000 per annum in the United States. However, they are rarely seen by ophthalmologists as these patients tend not to experience visual symptoms [1]. Metastases to the choroid are typically pale yellow in colour with the exception of thyroid, renal cell, and carcinoid metastases, which are known to have an orange appearance [2]. Hence an isolated choroidal metastasis from thyroid gland carcinoma can appear similar to a circumscribed choroidal haemangioma. In addition, choroidal metastases are usually associated with increased subretinal fluid and a retinal detachment. However, this patient did not present with a detachment. In this case, the dramatic change in appearance of the choroidal mass following treatment for stage 4 follicular cell carcinoma indicated that the mass was a metastatic deposit.

The 5-year relative survival rates for thyroid cancer have increased from 93% in 1983–1985 to 97% in 1995–2001 because of current treatment regimes [3]. Treatment consists of a combination of surgery, radioactive iodine treatment, and thyroid hormone replacement. ^131^I ablation targets both normal thymocytes and thyroid cancer cells which are characterized by possession of a sodium iodide symporter. They have therefore the ability to concentrate beta-emitting radiolabeled iodine. Therefore, ^131^I is largely specific for the target cancer cell. In addition, the emission of *γ*-rays suitable for imaging the drug distribution [4] enables whole-body scans enhancing the sensitivity of subsequent surveillance and potentially decreasing recurrence rate and mortality [5].

The early response of the choroidal metastasis to radioactive iodine is interesting. Ocular metastases may respond to systemic treatment such as chemotherapy but they may take many months to change in appearance. This dramatic, rapid fibrosis of a choroidal metastasis has, to our knowledge, not been described previously following ^131^I ablation treatment of follicular cell carcinoma of the thyroid gland.

Different types of thyroid carcinoma have been associated with the development of choroidal metastases such as papillary, follicular cell, Hurthle cell, and medullary cell carcinoma [6]. Following a review of the literature [7–12], 6 cases of follicular carcinoma metastatic to the choroid have been described (Table 1). In all cases, including the present case, patients presented with coexisting metastases in bone or lung; therefore the eye is not an early metastatic site. Median age of the patient was 74 years (range 50–83 years) with no sex predilection. Initial symptoms reported were loss of visual acuity, loss of visual field, and dyschromatopsia though one case was asymptomatic. The extent of retinal detachment reported varied from a minimal serous detachment to a total retinal detachment. In our case no serous fluid was detected. In 5 of the 6 cases published the lesion was a unilateral, isolated metastatic deposit.

In summary, metastatic thyroid cancer to the choroid can appear similar to circumscribed choroidal haemangioma. Response to systemic treatment with radiolabeled iodine was dramatic and produced complete regression of the choroidal lesion within 3 months. No local ocular treatment was required.

## Figures and Tables

**Table 1:** Previously reported cases of choroidal metastases secondary to follicular cell thyroid cancer (FTC).

Reference	Age, sex	Presentation	Fundus	Location of concurrent metastases	Time from initial diagnosis	Treatment
Ritland et al. [11]	80, f	Lateral visual field loss	Orange brown tumour nasally	Lung, bone, and mediastinum	40 yrs	^ 131^I
Scott et al. [9]	50, m	Blurred vision metamorphopsia	5.6 mm in elevation 12.9 × 11	Lung and bone	5 yrs	RTx, chemotherapy, and bevacizumab
Slamovits et al. [12]	64, f	Dyschromatopsia, total retinal detachment following vitreous haemorrhage	Pink mass, radioactive iodine accumulation	Paratracheal, bone, and lung	7 yrs	RTx
Arat and Boniuk [10]	83, m	Bilateral progressive loss of vision	Iris and bilateral choroidal lesions. Right eye: 2 orange-coloured lesions. Left eye: large reddish vascular lesion with subretinal haem	Lung, bone, liver, and skin	Initial presentation	^ 131^I
Seneviratne et al. [8]	74, f	Asymptomatic	Large yellow lesion in periphery	Lung, bone, and abdominal wall	22 yrs	RTx
Guignier et al. [7]	75, m	Decline in visual acuity	Amelanotic lesion with serous detachment	N/A	N/A	RTx

vf: visual field, RTx: radiotherapy.
